# Insights from rare variants into the genetic architecture and biology of youth-onset type 2 diabetes

**DOI:** 10.21203/rs.3.rs-2886343/v1

**Published:** 2023-05-18

**Authors:** Soo Heon Kwak, Shylaja Srinivasan, Ling Chen, Jennifer Todd, Josep Mercader, Elizabeth Jensen, Jasmin Divers, Amy Mottl, Catherine Pihoker, Rachelle Gandica, Lori Laffel, Elvira Isganaitis, Morey Haymond, Lynne Levitsky, Toni Pollin, Jose Florez, Jason Flannick

**Affiliations:** Seoul National University College of Medicine; University of California at San Francisco; Diabetes Research Center (Diabetes Unit), Department of Medicine, Massachusetts General Hospital; University of Vermont; Broad Institute; Wake Forest School of Medicine; NYU Langone Health; University of North Carolina; Seattle Children’s Hospital; Columbia University Irving Medical Center; Joslin Diabetes Center; Joslin Diabetes Center; Baylor College of Medicine; Massachusetts General Hospital for Children; University of Maryland School of Medicine; Massachusetts General Hospital; Broad Institute of MIT and Harvard/Boston Children’s Hospital/Harvard Medical School

## Abstract

Youth-onset type 2 diabetes (T2D) is a growing public health concern. Its genetic basis and relationship to other forms of diabetes are largely unknown. To gain insight into the genetic architecture and biology of youth-onset T2D, we analyzed exome sequences of 3,005 youth-onset T2D cases and 9,777 ancestry matched adult controls. We identified (a) monogenic diabetes variants in 2.1% of individuals; (b) two exome-wide significant (*P* < 4.3×10^−7^) common coding variant associations (in *WFS1* and *SLC30A8*); (c) three exome-wide significant (*P* < 2.5×10^−6^) rare variant gene-level associations (*HNF1A*, *MC4R*, *ATX2NL*); and (d) rare variant association enrichments within 25 gene sets broadly related to obesity, monogenic diabetes, and β-cell function. Many association signals were shared between youth-onset and adult-onset T2D but had larger effects for youth-onset T2D risk (1.18-fold increase for common variants and 2.86-fold increase for rare variants). Both common and rare variant associations contributed more to youth-onset T2D liability variance than they did to adult-onset T2D, but the relative increase was larger for rare variant associations (5.0-fold) than for common variant associations (3.4-fold). Youth-onset T2D cases showed phenotypic differences depending on whether their genetic risk was driven by common variants (primarily related to insulin resistance) or rare variants (primarily related to β-cell dysfunction). These data paint a picture of youth-onset T2D as a disease genetically similar to both monogenic diabetes and adult-onset T2D, in which genetic heterogeneity might be used to sub-classify patients for different treatment strategies.

Although type 2 diabetes (T2D) is most commonly diagnosed after age 40^1^, there are rare cases where children or adolescents (age < 20) develop T2D (hereafter youth-onset T2D)^2,3^. The prevalence of youth-onset T2D nearly doubled between 2001 and 2017^4^ in the United States, impacting personal and public health due to increased and earlier onset of diabetes-related complications^5^, most of which disproportionately affect minority race and ethnic groups^5^. Youth-onset T2D is strongly influenced by genetic factors^6–8^ and is characterized by both increased insulin resistance, as is adult-onset T2D, and rapidly progressive β-cell failure, as in certain types of early-onset monogenic diabetes^9^. Therefore, as for other complex diseases with clinically similar early-onset forms^10,11^, adult-onset T2D, youth-onset T2D, and monogenic diabetes are on a phenotypic spectrum. The pathophysiological and genetic relationships along the spectrum, however, are unclear^8^.

There are three logical hypotheses regarding the genetic variants, genes, and pathways involved in youth-onset T2D susceptibility. First, some youth-onset T2D risk variants might impact the same genes and pathways involved in adult-onset T2D, causing younger onset due to larger effect sizes^12^ or to an increased polygenic burden of variants relative to adult-onset T2D^2^. Second, some risk variants may impact the same pathways that cause monogenic diabetes but have a reduced penetrance relative to variants that cause monogenic diabetes^13^. Finally, some risk variants may lie in genes or pathways unique to youth-onset T2D. Understanding the degree to which these hypotheses explain youth-onset T2D, both at the population and individual levels, would have implications not only for our understanding of diabetes but also for understanding early-onset forms of other complex diseases.

Although much is known about common^14^ and rare^13^ variants that contribute to adult-onset T2D, as well as genes involved in monogenic diabetes^15^, genetic studies of youth-onset T2D have been limited. Two recent studies from the Progress in Diabetes Genetics in Youth (ProDiGY) consortium probed the role of common and monogenic variants in youth-onset T2D, with findings that suggested: (a) an elevated polygenic risk for adult-onset T2D-associated common variants is partially responsible for the risk of youth-onset T2D^2^, and (b) pathogenic or likely pathogenic variants within 11 genes for monogenic diabetes explain about 2–3% of youth-onset T2D cases^3^. However, neither study comprehensively analyzed the spectrum of coding alleles across the genome. In our present study, we analyzed whole-exome sequence data from these same ProDiGY samples to identify rare variant gene-level associations that could further elucidate the genetic and biological basis of youth-onset T2D and evaluate their contribution to youth-onset T2D relative to common variants.

## Genetic discovery

**Figure 1:**
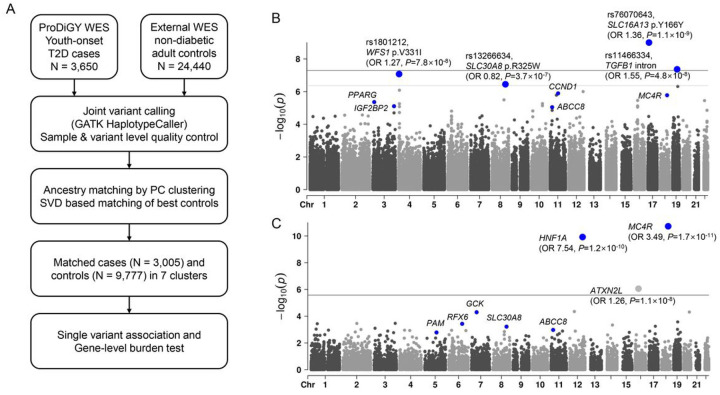
Scheme of the study and genetic discovery. **a.** Whole-exome sequence data of youth-onset T2D cases were matched to that of external non-diabetic controls using genetic PCs and a SVD based method resulting in 3,005 cases and 9,777 controls for single variant and gene-level association analysis. **b.** Single variant association analysis revealed four variants passing exome wide significance (*P* < 4.3 × 10^− 7^). **c.** Gene-level association analysis showed three genes associated with youth-onset T2D at exome-wide significance (*P* < 2.6 × 10^−6^). Blue dots represent previously known variants or genes of T2D. GATK, genome analysis tool kit; OR, odds ratio; PC, principal componants; SVD, singular value decomposition; WES, whole-exome sequencing.

We obtained 3,650 exome sequences from the ProDiGY Consortium, which consists of youth-onset T2D cases from five ancestry groups and three studies (Fig. 1A, **Methods**): SEARCH for Diabetes in Youth (SEARCH, N = 553; an observational study which enrolled youth diagnosed with T2D at age < 20 years^16^), Treatment Options for Type 2 Diabetes in Adolescents and Youth (TODAY, N = 526; a clinical trial investigating therapeutic strategies for T2D in overweight/obese youth aged 10–17^17^), and TODAY Genetics (N = 2,571; an ancillary study of TODAY with similar inclusion criteria). We combined the ProDiGY data with exome sequences from 24,440 non-diabetic adult controls, spanning five ancestry groups from the recent AMP-T2D-GENES T2D exome sequencing study^13^. After joint variant calling and quality control (**Methods**), we used a combination of genetic principal component (PC) clustering (**Supplementary Fig. 1**) and a singular value decomposition (SVD) based method^18^ (**Methods**) to genetically match 3,005 youth-onset T2D cases to 9,777 controls (effective sample size 9,194). Our final analysis set included samples from seven PC-based clusters and three major ancestries (African American: N = 4,189; Europeans: N = 2,546; Hispanics: N = 6,047; **Supplementary Table 1**) and contained 670,704 variants that passed quality control filters in the coding region (246,694 non-synonymous) across 17,950 genes. As expected, the youth-onset T2D cases were younger (mean age at diagnosis 13.6 ± 2.4) and had a higher BMI (Z-score 2.2 ± 0.6) than did their ancestry matched controls (age 54.4 ± 11.6 years and BMI Z-score 1.2 ± 0.6; **Supplementary Table 1**).

To identify genetic risk factors for youth-onset T2D, we performed single-variant and gene-level rare variant association analyses following a previously developed quality control and regression procedure (**Methods**); for gene-level tests, we analyzed seven nested ‘masks’ of variant sets and summarized results for each gene into a single *P* value corrected for the effective number of tests for the gene^19^. Neither single-variant nor gene-level associations showed any evidence of systemic test-statistic inflation (**Supplementary Figs. 2 and 3**), and gene-level association tests of synonymous variants produced no significant associations (**Supplementary Fig. 4**), suggesting that our strategy to incorporate external control exomes produced well-calibrated test-statistics.

Two single variants produced genome-wide significant (*P* < 5.0×10^− 8^) association with youth-onset T2D: rs76070643, a synonymous variant in *SLC16A13*, and rs11466334, an African-specific intronic variant of *TGFB1* (Fig. 1B, **Supplementary Table 2**). Two additional nonsynonymous variants produced associations significant under a more lenient exome-wide significance threshold^20^ (*P* < 4.3×10^− 7^) (**Methods**): rs1801212 in *WFS1*, and rs13266634 in *SLC30A8*. All four variants were previously reported as associated with adult-onset T2D^21^. However, all had larger effect sizes in youth-onset T2D than in adult-onset T2D (a mean OR increase of 1.14-fold; **Supplementary Table 3**).

Three gene-level associations (*MC4R*, *HNF1A*, and *ATXN2L*) attained exome-wide significance (*P* < 2.6×10^− 6^; Fig. 1C, **Supplementary Table 4**). For both *MC4R* (OR 3.49, 95% confidence interval [CI] 2.43–5.02, *P* = 1.7×10^− 11^, combined minor allele frequency [MAF] 0.011 for 25 variants) and *HNF1A* (OR 7.54, 95% CI 4.08–13.9, *P* = 1.2×10^− 10^, combined MAF 0.0038 for 21 variants), multiple variants contributed to the associations, multiple masks produced significant associations, and effect sizes were larger for more stringent masks that contained a greater proportion of predicted damaging variants (**Supplementary Fig. 5–7**). *MC4R* is recognized for harboring rare and common variants associated with obesity^22,23^ and T2D^13,24^; however, its rare variant association with youth-onset T2D had a 1.75-fold larger effect size and was an order of magnitude more significant than previously observed for adult-onset T2D^13^ (adult-onset OR 2.07, *P* = 2.7×10^− 10^, N = 20,791 cases). The youth-onset gene-level association was partly attributed to three rare variants that each individually reached nominal significance (*P* < 0.05) and had larger effect sizes for youth-onset T2D than for adult-onset T2D (p.I269N OR 2.93 vs 2.17, p.P299H OR 16.82 vs 1.97, and p.T150I OR 7.30 vs 2.31). Similar to *MC4R*, *HNF1A* harbors variants that cause maturity-onset diabetes of the young (MODY^25^) as well as rare and common variants associated with adult-onset T2D^26^. Its rare variant gene-level association with youth-onset T2D had a six-fold greater effect size and was eight orders of magnitude more significant than previously reported for adult-onset T2D (adult-onset OR 1.23, *P* = 0.022, N = 20,791 cases). Both the *HNF1A* and *MC4R* associations spanned multiple ancestries, demonstrating large effect sizes (OR > 3) and reaching nominal significance in African American, European, and Hispanic youth-onset T2D cases (**Supplementary Table 5**).

The third exome-wide significant gene-level association was observed for *ATXN2L* (OR 1.26, 95% CI 1.15–1.39, *P* = 1.1×10^− 6^, combined MAF 0.36 for 73 variants). Although it lies near GWAS associations for multiple obesity phenotypes^27^, *ATXN2L* has no confirmed role in the etiology of diabetes or obesity. The gene-level *ATXN2L* association was primarily due to a single common variant (rs55719896, *P* = 5.7×10^− 5^) within an *ATXN2L* splice acceptor site. This variant is known for its association with obesity and diabetes^28^, and has allele-specific expression in pancreatic islets (http://tiger.bsc.es)^29^. However, its causal relationship requires further investigation, as it is in strong linkage disequilibrium (R^2^ = 0.99) with an intronic variant (rs9972768) of *SH2B1*, a gene with a confirmed role in BMI variability^27^. Nevertheless, the *ATXN2L* gene-level association did include an additional 72 rare variants that strengthened its significance by more than two orders of magnitude (*P* value decreasing to 2.4×10^− 7^, **Supplementary.Fig. 7**)

The overlap of these youth-onset T2D associations with those previously identified for adult-onset T2D supports a model in which youth-onset T2D cases harbor genetic risk factors that also predispose to adult-onset T2D. To investigate this model further, we assembled a broader collection of adult-onset T2D-associated common coding variants and evaluated their association with youth-onset T2D. Among the 17 single variants (15 of which had MAF ≥ 5%) with the strongest adult-onset T2D associations (*P* < 1.0×10^− 5^) in the previous AMP-T2D-GENES^13^ exome analysis, 13 showed nominal (*P* < 0.05) associations with youth-onset T2D in ProDiGY, all of which had consistent directions of effect for adult-onset and youth-onset T2D and all of which had larger effect sizes in ProDiGY than in AMP-T2D-GENES (binomial *P* < 0.0001 accounting for control sample overlap, sample size difference, and winner’s curse, **Methods**, **Supplementary Table 6**). Similarly, of the 40 coding variants with the strongest adult-onset T2D associations in the DIAGRAM consortium^30^, 35 were available and 31 had consistent directions of effect (binomial *P* < 0.0001) and 25 had larger effect sizes (binomial *P* < 0.0001, average 1.18-fold increase; **Supplementary Table 7**) in ProDiGY.

Regarding gene-level associations, among the 50 with the strongest adult-onset T2D associations (*P* < 2.0 × 10^− 3^) in AMP-T2D-GENES^13^, 71.7% (33 of 46 available in ProDiGY) showed a consistent direction of effect in ProDiGY and 54.5% of these (18 of 33) had larger effect sizes in ProDiGY (binomial *P* < 0.0001 for having both consistent direction and larger effect, considering sample size difference, overlap of controls, and winner’s curse^31^, **Supplementary Table 8**). Similarly, we curated a “known diabetes gene set” (including monogenic diabetes, T2D drug targets, T2D GWAS genes with causal coding variants, monogenic obesity, and mouse diabetes, **Methods**, **Supplementary Table 9**) among which 38 genes had nominal (*P* < 0.05) association in either AMP-T2D-GENES or ProDiGY. Of these 38 genes, 27 had consistent directions of effect between the two studies, and 21 of these (77.8%) had larger effect sizes in ProDiGY than in AMP-T2D-GENES (expected frequency of 60% based on sample size difference and overlap of controls, binomial *P* < 0.016, 2.86-fold average increase; **Supplementary Table 10**). Thus, the strongest adult-onset T2D genetic risk factors (both common and rare variants) were also observed in youth-onset T2D cases, at a greater frequency in youth-onset compared to adult-onset T2D cases.

We further tested whether youth-onset T2D cases are enriched for risk factors for other forms of diabetes or related phenotypes, including monogenic diabetes (MODY and neonatal diabetes), syndromic diabetes, monogenic obesity, lipodystrophies, and type 1 diabetes (**Supplementary Table 11**). Wilcoxon rank-sum tests of ProDiGY associations within six gene sets curated for these phenotypes (**Methods**) produced four significant gene set associations (*P* < 0.05). The strongest association was observed for a ‘Monogenic OMIM’ gene set with 13 genes for MODY and neonatal diabetes (7.03-fold enrichment, *P* = 8.7×10^− 6^). A gene set of 37 monogenic obesity genes also showed a nominally significant gene set enrichment in ProDiGY (2.05-fold enrichment, *P* = 0.034). The other two significant associations (‘Monogenic primary’, 27 genes with variants causing diabetes as a primary phenotype, and ‘Monogenic all’, 80 genes with variants causing monogenic or syndromic diabetes or affecting glycemic traits) showed significant associations only because they subsumed the ‘Monogenic OMIM’ gene set (gene set *P* > 0.25 after removing ‘Monogenic OMIM’ genes). This result, together with the lack of a significant association for a lipodystrophy gene set (*P* > 0.50), suggests that youth-onset T2D is unlikely to primarily represent a collection of rare syndromic forms of diabetes. A type 1 diabetes gene set also did not show a significant ProDiGY association (*P* > 0.50), suggesting that youth-onset T2D shares genetic variants and genes overlapping those for some, but not all, forms of diabetes.

## Insights into youth-onset T2D pathophysiology

**Figure 2:**
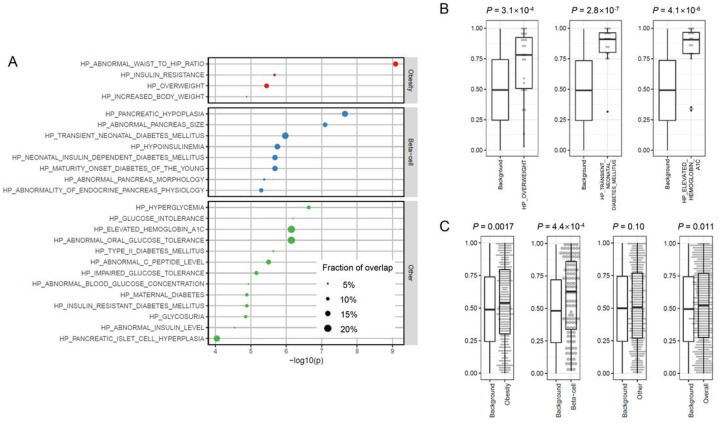
Pathways involved in obesity and β-cell function are enriched in youth-onset T2D. **a.** Gene set enrichment analysis using hypergeometric test with top 50 gene-level association signals in youth-onset T2D identified 25 Human Phenotype Ontology gene sets which had significant overlap and were related to metabolic phenotypes of diabetes. These 25 gene sets were categorized into three subgroups of ‘obesity’, ‘β-cell function’, and ‘others’. **b.** Wilcoxon rank-sum test using these 25 gene sets revealed representative sets with significant association enrichments beyond the top 50 associated genes, such as ‘HP_OVERWEIGHT’, ‘HP_TRANSIENT_NEONATAL_DIABETES_MELLITUS’, and ‘HP_ELEVATED_HEMOGLOBIN_A1C’. **c.** Gene set clusters of ‘obesity’ and ‘β-cell function’ showed significant enrichment (*P* < 0.05) when combining genes across all sets in the cluster. Background denotes matched genes with similar numbers and frequencies of variants within them.

To further elucidate the genes and pathophysiology involved in youth-onset T2D, we extended our gene set enrichment analysis to 5,071 gene sets defined by Human Phenotype Ontology^32^ terms curated by the Molecular Signature Database^33,34^ (**Methods**). We first focused on the 50 most significant ProDiGY gene-level associations – a threshold above which ProDiGY associations showed excess replication in the AMP-T2D-GENES study (**Supplementary Table 12, Supplementary Fig. 8**). A gene set enrichment analysis showed these “top 50 genes” to be over-represented in 38 gene sets (at false discovery rate q-value < 0.01; **Supplementary Table 13**), 25 of which were defined using terms related to metabolic phenotypes of T2D (e.g., ‘diabetes’, ‘hyperglycemia’, ‘overweight’, ‘waist’, ‘insulin’, ‘c-peptide’, **Methods**). These 25 enriched gene sets further clustered into three subgroups (Fig. 2A): ‘obesity’ (e.g. ‘HP_ABNORMAL_WAIST_TO_HIP_RATIO’), ‘β-cell function’ (e.g. ‘HP_PANCREATIC_HYPOPLASIA’), and ‘other T2D’ (e.g. ‘HP_HYPERGLYCEMIA’). We found similar results when we repeated this analysis with smaller (N = 25) or larger (N = 100) lists of top genes (**Supplementary Table 14–15**).

To evaluate if these 25 enriched metabolic gene sets contained additional genes (beyond the “top 50”) that might be involved in youth-onset T2D, for each gene set we applied a Wilcoxon rank-sum test to the *P*-values of all genes in the set (**Methods**). Among the 25 gene sets, 14 were significantly enriched for ProDiGY associations after multiple test correction (Wilcoxon *P* < 0.002) and 21 were enriched at nominal significance (*P* < 0.05) (**Supplementary Table 16**). Gene sets in each of the three clusters showed significant enrichments, led by ‘HP_OVERWEIGHT’ in the ‘obesity’ cluster (*P* = 3.1×10^− 4^, driven by 7 genes), ‘HP_TRANSIENT_NEONATAL_DIABETES_MELLITUS’ in the ‘β-cell function’ cluster (*P* = 2.8×10^− 7^, driven by 10 genes), and ‘HP_ELEVATED_HEMOGLOBIN_A1C’ in the ‘other T2D’ cluster (*P* = 4.1×10^− 6^, driven by 8 genes) (Fig. 2B). The ‘obesity’ and ‘β-cell function’ clusters (but not the ‘other T2D’ cluster) in fact showed significant enrichments (*P* < 0.05) when we combined genes across all sets in the cluster (Fig. 2C). Even after removing the top 50 genes from this analysis, 16 gene sets remained significant at *P* < 0.05 and 7 remained significant at *P* < 0.002, providing evidence for rare variant youth-onset T2D risk factors spread across many genes and falling short of exome-wide significance in our current analysis (**Supplementary Table 17**).

We used the ProDiGY rare variant gene-level and gene set associations to define four tiers of genes with potential rare variant risk factors for youth-onset T2D (**Methods**, **Supplementary Table 18**). “Tier 1” contains the three genes with exome-wide significant ProDiGY gene-level associations (*MC4R*, *HNF1A*, and *ATXN2L*). “Tier 2” additionally includes four genes within the top 50 genes and either being causal for monogenic diabetes or harboring causal coding variants of T2D^35^ (*GCK*, *SLC30A8*, *ABCC8*, and *PAM*). “Tier 3” additionally includes four genes among the top 50 genes and within at least two of the 25 significantly enriched gene sets with metabolic phenotypes of T2D (*RFX6*, *GHRL*, *HESX1*, and *SIX3*), bringing the total number of genes in this tier to 11. Finally, a more lenient “Tier 4” includes 46 genes that were nominally significant (*P* < 0.05) among 25 gene sets that showed enrichment and were related to metabolic phenotypes of T2D.

Focusing on “Tier 3” for our primary analysis (**Methods**), we divided its 11 genes into three groups: “obesity-related”, “monogenic diabetes”, and “β-cell-related” genes, based on 1) prior knowledge, 2) their gene set annotations, and 3) their proximity to GWAS associations (**Supplementary Table 19**). The “obesity-related” gene group contains *MC4R*, *ATXN2L*, *GHRL (*OR 1.99, *P* = 8.0×10^− 4^) and *HESX1* (OR 0.23, *P* = 2.6×10^− 3^). *GHRL* encodes ghrelin, one of the most powerful appetite stimulating hormones^36^, plasma levels of which are associated with adiposity and growth hormone release^37^. *HESX1* encodes a homeobox protein that acts as a transcriptional repressor in the pituitary gland. Pathogenic variants of *HESX1* are associated with pituitary hormone deficiency and septo-optic dysplasia and may be associated with obesity^38,39^.

The “monogenic diabetes” gene group contains *HNF1A*, *GCK*, *RFX6*, and *ABCC8*, which harbor variants that cause MODY. Like *HNF1A*, both *GCK* (OR 7.69, *P* = 5.0×10^− 5^) and *RFX6* (OR 47.9, *P* = 3.7×10^− 4^) had ProDiGY associations with large effect sizes, 3.1-fold and 22-fold higher (respectively) than their association effect sizes in adult-onset T2D (AMP-T2D-GENES). In *RFX6*, six heterozygous singleton variants (one splice acceptor, two stop codon, and three missense variants) contributed to the ProDiGY youth-onset T2D association. This suggests that missense in addition to protein-truncating variants (PTVs) of *RFX6* could contribute to diabetes in the heterozygous state, expanding the *RFX6* allelic series^40^. In *ABCC8*, the observed ProDiGY youth-onset T2D association (OR 1.46, *P* = 1.1×10^− 3^) stood in stark contrast to the previously reported lack of adult-onset T2D association in AMP-T2D-GENES (OR 0.96, *P* = 0.96)^13^.

The “β-cell-related” gene group includes *SLC30A8*, *SIX3*, and *PAM*, genes involved in pancreatic β-cell function but not monogenic diabetes. *SIX3* (OR 6.96, *P* = 0.0026) is a critical transcription factor expressed in human β-cells after childhood^41^ and is involved in insulin secretion^42^. Decreased activity of *PAM* (OR 1.42, *P* = 0.0017) results in impaired insulin secretion in human pancreatic islets^43^, and *SLC30A8* (OR 0.35, *P* = 6.0×10^− 4^) encodes a zinc transporter within pancreatic islet insulin secretory granules^44^. Each of these three genes lies near or harbors variants associated with adult-onset T2D^30,45^, and *SLC30A8* carries further loss-of-function and missense mutations that enhance insulin secretion^46^ and protect against adult-onset T2D^13,47^. The protective *SLC30A8* association we observed with youth-onset T2D is noteworthy, as it suggests that youth-onset disease risk can be influenced not only by the presence of strong-effect variants that increase risk of disease, but also (in a permissive environmental and genetic context) by a depletion of variants that protect against disease.

## Population-level genetic architecture of youth-onset T2D

As our analyses suggested that monogenic, rare, and common variants each play a role in youth-onset T2D, we sought to compare the relative contribution of these risk factors to youth-onset T2D at the population-level. We first examined variants in the 13 known MODY or neonatal diabetes genes, five of which (*HNF1A*, *GCK*, *ABCC8*, *INS*, and *KLF11*) were associated with increased risk of youth-onset T2D at nominal significance (*P* < 0.05) in ProDiGY (**Supplementary Table 20**). As reported previously^3^, these associations were due in part to (presumably undiagnosed) MODY cases within ProDiGY: 62 (2.1%) of youth-onset T2D cases carried variants in these genes classified as pathogenic or likely pathogenic, the majority within *HNF1A* (32 cases) and *GCK* (16 cases). However, even after removing these 48 MODY variant carriers, the association of *HNF1A* (OR 4.82, *P* = 1.1 × 10^− 5^) and *GCK* (OR 2.83, *P* = 9.2 × 10^− 3^) remained significant (**Supplementary Table 21**). These data support an expansion of the allelic series in MODY genes to include not only variants that cause MODY^26^ and risk factors for adult-onset T2D^28^ but also risk factors for youth-onset T2D.

**Figure 3:**
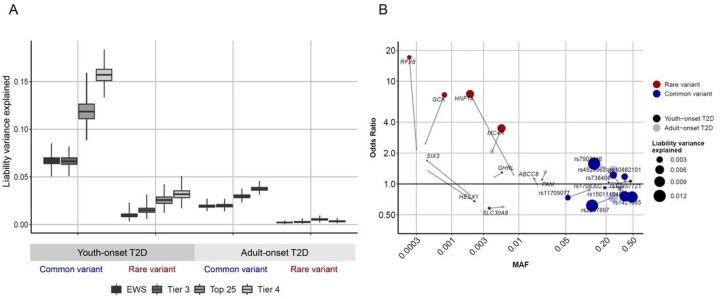
Genetic architecture and liability variance explained by common and rare variants. **a.** Liability variance explained by common variants and gene-level associations in youth-onset T2D and adult-onset T2D for exome-wide significant associations (EWS), 10 Tier 3 genes and same number of common variants (Tier 3), top 25 significant gene-level and common variant associations (Top 25), and 46 Tier 4 genes and same number of common variants (Tier 4). The liability variance explained by common variants increased by 3.5 – 4.2-fold in youth-onset T2D compared to adult-onset T2D. There was even larger 5.0 – 9.0-fold increase in liability variance explained by rare variant gene-level associations in youth-onset T2D. **b.** Odds ratio and allele frequency distribution of Tier 3 gene-level and common variant association signals and their liability variance explained in youth-onset T2D and adult-onset T2D.

We next evaluated, beyond variants in monogenic diabetes genes, the fraction of youth-onset T2D liability variance explained (LVE) by rare and common variants (after correcting for winner’s curse and other confounders, **Methods**). Considering the 10 gene-level rare variant associations within “Tier 3” genes (we excluded *ATXN2L*, whose association is driven by a common variant) and the 10 strongest ProDiGY common variant associations, we noted 4.5-fold larger LVE (within ProDiGY) by common variant associations compared to rare variant gene-level associations (6.7%, 95% CI 6.3–7.0 vs 1.5%, 95% CI 1.3–1.7) (Fig. 3A, **Supplementary Table 22**). This observation was consistent across comparisons of different sets of common and rare variant associations, including exome-wide significant associations (6.7% vs 1.0%), the 25 strongest gene-level and 25 strongest common variant associations (11.9% vs 2.6%), or “Tier 4” genes and the same number of common variant associations (15.7% vs 3.2%) (**Supplementary Table 23–25**). Across these analyses, common variant LVE was substantially higher (3 to 7-fold) than the percentage of cases explained by MODY variants, and rare variant LVE was roughly the same (0.5-fold to 2-fold higher depending on the analysis). This dominant contribution to LVE from common variants suggests that the genetic architecture of youth-onset T2D resembles that for adult-onset T2D more so than it resembles that for a monogenic disease.

To further compare the genetic architectures of youth-onset and adult-onset T2D, we computed the LVE by the 10 strongest common variant associations and 10 “Tier 3” gene-level associations in ProDiGY and AMP-T2D-GENES (correcting for winner’s curse, different sample sizes, and control sample overlap; **Methods**). The common variant LVE was 3.4-fold larger (*P* < 0.0001) for youth-onset T2D (6.7%, 95% CI 6.3–7.0) than for adult-onset T2D (2.0%, 95% CI 1.9–2.1; **Supplementary Table 26**), and the rare variant LVE was 5.0-fold larger (*P* < 0.0001) for youth-onset T2D (1.5%, 95% CI 1.3–1.7) compared to adult-onset T2D (0.3%, 95% CI 0.2–0.3). These trends of 1) larger absolute LVE by both common variants and rare variants in ProDiGY relative to AMP-T2D-GENES and 2) a greater fold-increase in rare variant LVE (5.0–9.0-fold increase) than common variant LVE (3.5–4.2-fold increase) in ProDiGY relative to AMP-T2D-GENES held across three alternative sets of gene-level and common variant associations and after adjustment for multiple potential confounders (**Methods**, **Supplementary Table 26**, Fig. 3B). Although these results apply only to the strongest observed associations in each of ProDiGY and AMP-T2D-GENES, they support a model in which youth-onset T2D is enriched for genetic risk factors of all frequencies – with stronger enrichments for rarer variants – compared to adult-onset T2D.

## Individual-level heterogeneity of youth-onset T2D

At the population-level, our data suggest that youth-onset T2D is influenced by common, rare, and monogenic variants. To investigate the heterogeneity of individual-level youth-onset T2D cases for these three classes of risk factor, we computed three “contribution scores” for each youth-onset T2D case (**Methods**): 1) a “rare variant score” of the combined ORs (after correction for winner’s curse) of variants within the 46 nominally significant (*P* < 0.05) genes of 25 gene sets that showed enrichment and were related to metabolic phenotypes of T2D as described above (tier 4 genes), 2) a “common variant score” of the combined ORs of 403 previously published genome-wide significant associations for T2D, and 3) a “combined score” defined as the sum of the two scores. We then identified and evaluated cases with high values (OR ≥ 5 or OR ≥ 3) for one or more of these contribution scores.

**Figure 4:**
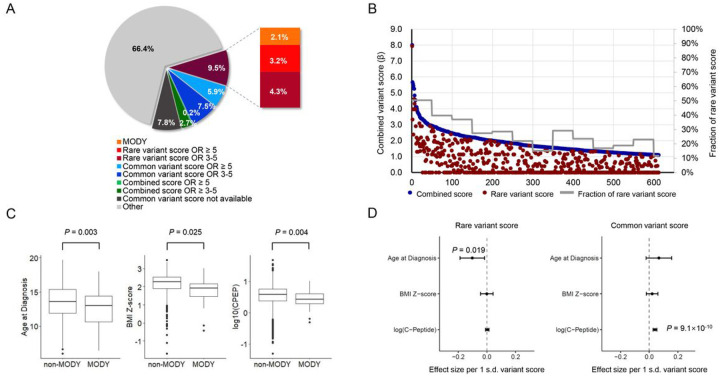
Individual genetic risk confered by common and rare variants. **a.** Fraction of individuals having high genetic risk confered by MODY variants, rare variant score, common variant score, or combined variant score. Among 3,005 youth-onset T2D cases, 2.1% carried MODY variants, 3.2% had high rare variant score with OR ≥ 5, 5.9% had high common variant score with OR ≥ 5, and 0.2% had high combined score with OR ≥ 5. **b.** For the 612 individuals having high combined variant score with OR ≥ 3, the contribution of rare variant score was higher at the higher end of the combined variant score. **c.** MODY cases had earlier age of diagnosis, lower BMI Z-score, and lower log_10_(C-peptide) level. **d.** Rare variant score was associated with earlier age at diagnosis and common variant score was associated with higher log_10_(C-peptide) level even after excluding MODY cases.

Among the 3,005 ProDiGY cases, 342 (11.4%) had either a MODY variant or OR ≥ 5 according to at least one of these contribution scores: 62 (2.1%) had a MODY variant, 96 (3.2%) had rare variant score OR ≥ 5, 177 (5.9%) had common variant score OR ≥ 5, and 7 (0.2%) had combined score (but not rare or common variant score) OR ≥ 5 (Fig. 4A). An additional 434 cases (14.4%) had youth-onset T2D OR between 3–5: 128 (4.3%) from the rare variant score, 224 (7.5%) from the common variant score, and 82 (2.7%) from the combined score. Although these ORs are only rough and partial estimates of each patient’s genetic risk, they suggest that youth-onset T2D cases exhibit individual-level heterogeneity in terms of which risk factors predominate.

To examine if this genetic heterogeneity enabled ProDiGY cases to be clustered into distinct subtypes, we identified 612 ProDiGY cases with 1) combined OR ≥ 3 and 2) both common and rare variant scores positive (OR > 1). Across these 612 cases, rare variant scores were on average 27.3% of the combined scores, consistent with the larger contribution of common variants to youth-onset T2D at the population-level. Among the cases with the highest combined scores, however, rare variant scores were on average 50.4% of the combined scores for the top 50 cases and on average 39.7% of the combined scores for the next 50 cases. This proportion steadily decreased as combined scores decreased, reaching 23.0% for individuals with ranks between 551–600 (Fig. 4B). This decrease was gradual, and there was no obvious boundary that could differentiate individuals with “high” *versus* “low” rare variant scores. Nonetheless, this broad trend supports a model in which more “extreme” youth-onset T2D cases are due more so to rare rather than common variants.

To finally evaluate whether a patient’s phenotypic presentation is influenced by the relative contribution of common or rare risk variants, or monogenic causes, we investigated the relationship between case genetic “contribution score” and case age at diabetes diagnosis, BMI, and C-peptide levels (a measure of insulin secretion). Cases with MODY variants had an earlier age of diagnosis (12.7 ± 2.5 vs 13.6 ± 2.3, *P* = 0.003), lower BMI Z-score (1.80 ± 0.83 vs 2.18 ± 0.57, *P* = 0.025), and lower log_10_(C-peptide) level (0.43 ± 0.26 vs 0.55 ± 0.33, *P* = 0.0011) than cases without MODY variants. Rare variant score (after removing MODY cases) was associated with earlier age at T2D diagnosis (β −0.102 years per 1 s.d. increase in risk score, *P* = 0.019, −0.3 years for cases in top 10% of rare variant score), but not with BMI Z-score or C-peptide level. (Fig. 4C, **Supplementary Table 27**). In contrast, common variant score was associated with higher log_10_(C-peptide) level (β = 0.040 per 1 s.d. increase in common variant score, *P* = 9.1×10^− 10^, 0.06 higher for cases in the top 10% of common variant score). We did not observe a significant trend in age of diagnosis across the entire distribution of common variant scores, but the 5% of cases with the highest scores did trend toward later onset (14.1 ± 2.3 vs 13.6 ± 2.3, *P* = 0.029) compared to the remaining cases (**Supplementary Table 28**). These findings support the hypothesis that MODY variants, rare variants, and common variants predispose to different presentations of youth-onset T2D, with common variants predisposing to a form of youth-onset T2D characterized by insulin resistance and rare variants predisposing to a leaner form of youth-onset T2D with an earlier age of onset.

## Discussion

Using whole-exome sequence data from 3,005 youth-onset T2D cases and 9,777 carefully matched external controls, we identified a variety of genetic risk factors with effect sizes much larger for youth-onset T2D than any observed for adult-onset T2D. A combination of gene-level and gene set associations prioritized 11 genes (grouped into three broad pathophysiological clusters) as potentially causal for youth-onset T2D, which enabled us to investigate the pathways involved in youth-onset T2D, its overlap with other forms of diabetes, and the role of different genetic risk factors in population risk and patient phenotypic presentation.

It is worth noting upfront some features of our study that qualify our conclusions below. First, while our sample size was large for a rare disease (and therefore permitted statistical rather than only deterministic analyses^48^), it was modest for a common disease and therefore required some of our inferences to include suggestive associations; in the context of population-level risk, we were additionally limited to evaluating contributions from the strongest, rather than all, genetic risk factors. Second, while we extensively validated our external control matching strategy (**Methods**), some population stratification likely persisted between cases and controls – particularly for rare variants^49^. Third, our comparisons between youth-onset and adult-onset T2D risk factors relied on the previous AMP-T2D-GENES study, which shared controls with our study; we employed extensive simulations and analytical calculations to control for this overlap – as well as winner’s curse in each study and their differential sample sizes (**Methods**) – but some biases in our estimates may remain. Therefore, our conclusions – although consistent across numerous sensitivity analyses (**Methods**) – are most robust when stated qualitatively. We first conclude that clinically diagnosed youth-onset T2D is influenced by – in order of importance – common variants, rare variants, and MODY variants. Youth-onset T2D cases appear enriched for each of these risk factors relative to adult-onset T2D cases – a result consistent with simulations of “extreme” common disease phenotypes^50^ – but their enrichment is sufficiently large that we identified more significant associations in ~ 3,000 youth-onset cases than were previously identified in ~ 20,000 adult-onset cases^13^. In relative terms, youth-onset T2D cases are more enriched for rare variant risk factors, a skew perhaps unsurprising for a rarer disease but one that is juxtaposed upon a background in which common variant risk factors still explain the most disease heritability. At the population level, youth-onset T2D therefore appears to share genetic features of both a common and rare disease.

We also conclude that youth-onset T2D is influenced by risk factors in multiple biological pathways, contributing to (at minimum) beta-cell development, insulin secretion, and obesity-related insulin resistance. These pathways overlap with those responsible for both adult-onset T2D and MODY but notably not all forms of diabetes – suggesting that youth-onset T2D is likely not a collection of syndromic diseases. Our analysis prioritized 11 genes as likely involved in youth-onset T2D – and 38 with some evidence for involvement in youth-onset T2D – with several interesting properties. For example, MODY genes showed association with youth-onset T2D even after pathogenic and likely pathogenic variants were removed from our analysis, expanding the known allelic series within these genes to include risk factors for youth T2D as well as variants causing MODY and risk factors for adult-onset T2D. We also identified suggestive associations in several obesity genes; while these associations warrant further replication and functional investigation, they contrast with findings from adult-onset T2D association studies that skew much more to genes involved in insulin secretion^51^. Also notable was a protective association between *SLC30A8* rare variants and youth-onset T2D, which had a larger effect than was observed for adult-onset T2D. This depletion of cases for protective variants contrasts with the usual model in which early-onset disease cases are due mostly to high-effect risk increasing variants.

Most intriguingly, the phenotype of individual youth-onset diabetes cases (age of onset, BMI and C-peptide level) seems to differ depending on whether genetic risk is due primarily to MODY variants, common variants, or rare variants. There are no clear dividing lines to classify individuals into different subtypes based on these risk classes, suggesting that youth-onset T2D genetic heterogeneity may be best considered a continuum. Larger sample sizes that enable further investigation into this heterogeneity – across not only variant frequencies but also pathways – may suggest a way to tailor genetic diagnosis, prediction, and precision therapy to patients with youth-onset T2D. This genetic model for youth-onset T2D may in turn help to better understand and categorize adult-onset and monogenic diabetes as well. It is likely that other diseases may have “intermediate” phenotypes^52^ whose analysis – by combining the strengths of rare and common disease analyses – may help illuminate the likely blurred line between phenotypically related disease forms.
